# The role of immunosuppressive myofibroblasts in the aging process and age-related diseases

**DOI:** 10.1007/s00109-023-02360-1

**Published:** 2023-08-22

**Authors:** Antero Salminen

**Affiliations:** Department of Neurology, Institute of Clinical Medicine, University of Eastern Finland, P.O. Box 1627, 70211 Kuopio, Finland

**Keywords:** Aging, CAF, Fibroaging, Fibrosis, Immunosuppression, Immunosenescence, Longevity

## Abstract

Tissue-resident fibroblasts are mesenchymal cells which control the structural integrity of the extracellular matrix (ECM). Fibroblasts possess a remarkable plasticity to allow them to adapt to the changes in the microenvironment and thus maintain tissue homeostasis. Several stresses, also those associated with the aging process, convert quiescent fibroblasts into myofibroblasts which not only display fibrogenic properties but also act as immune regulators cooperating both with tissue-resident immune cells and those immune cells recruited into affected tissues. TGF-β cytokine and reactive oxygen species (ROS) are major inducers of myofibroblast differentiation in pathological conditions either from quiescent fibroblasts or via transdifferentiation from certain other cell types, e.g., macrophages, adipocytes, pericytes, and endothelial cells. Intriguingly, TGF-β and ROS are also important signaling mediators between immunosuppressive cells, such as MDSCs, Tregs, and M2 macrophages. It seems that in pathological states, myofibroblasts are able to interact with the immunosuppressive network. There is clear evidence that a low-grade chronic inflammatory state in aging tissues is counteracted by activation of compensatory immunosuppression. Interestingly, common enhancers of the aging process, such as oxidative stress, loss of DNA integrity, and inflammatory insults, are inducers of myofibroblasts, whereas anti-aging treatments with metformin and rapamycin suppress the differentiation of myofibroblasts and thus prevent age-related tissue fibrosis. I will examine the reciprocal interactions between myofibroblasts and immunosuppressive cells within aging tissues. It seems that the differentiation of myofibroblasts with age-related harmful stresses enhances the activity of the immunosuppressive network which promotes tissue fibrosis and degeneration in elderly individuals.

## Introduction

**Fig. 1:**
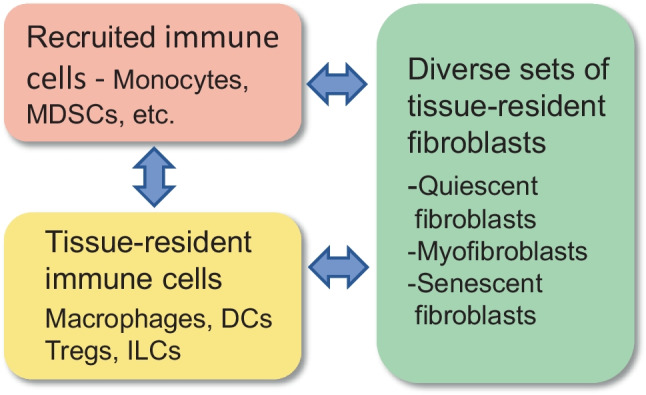
Interactions between fibroblasts and immune cells in aging tissues. Fibroblasts are a heterogeneous group of cells involving quiescent fibroblasts, activated myofibroblasts, and senescent fibroblasts. The immune cell population includes the tissue-resident cells, such as macrophages, DCs, Tregs, and ILCs, and the tissue-recruited immune cells, e.g., monocytes, macrophages, and MDSCs. Myofibroblasts can activate tissue-resident immune cells as well as recruiting immune cells into tissues. There is a reciprocal cooperation between fibroblasts and immune cells since immune cells, especially immunosuppressive cells, can modify the activities of fibroblasts. Abbreviations: DC, dendritic cell; ILC, innate lymphoid cell; MDSC, myeloid-derived suppressor cell; Treg, regulatory T cell

Tissue-resident fibroblasts maintain the integrity of connective tissues by secreting the proteins of the extracellular matrix (ECM) and, accordingly, producing the proteolytic enzymes which degrade the components of the ECM. Fibroblasts possess a remarkable phenotypic plasticity since the cells of host tissues and infiltrated immune cells can modulate the properties of fibroblasts, especially in pathological conditions [1] (Fig. 1). Moreover, under harmful conditions, not only tissue-resident fibroblasts differentiate into myofibroblast but also many other cell types, e.g., inflammatory macrophages, adipocytes, pericytes, and smooth muscle cells, can transdifferentiate into myofibroblasts [2]. Myofibroblasts are fibrogenic cells which promote the process of fibrosis in several pathological states including age-related fibrosis in many tissues [3–5]. Interestingly, fibroblasts, especially the myofibroblasts, can act as immune regulators displaying either pro-inflammatory or immunosuppressive properties. Cancer-associated fibroblasts (CAF) are able to exhibit both inflammatory phenotypes (iCAF) and myofibroblastic features (myCAF). The myCAFs possess many immunosuppressive properties which can enhance tumor growth [6]. Tissue-resident and cultured fibroblasts are also able to switch to a state of cellular senescence similarly as that encountered in many other cell types [7]. Considering the plasticity of fibroblasts and their close collaboration with immune cells, it seems evident that tissue fibroblasts possess many of the properties linked with the promotion of the aging process.

The aging process is associated with the presence of a low-grade chronic inflammation in the tissues, termed as inflammaging [8]. Currently, the primary cause of the inflammaging state in tissues needs to be clarified although it is known that with aging there is an accumulation of senescent cells within tissues where they secrete pro-inflammatory factors, such as cytokines and chemokines [9]. Subsequently, these inflammatory mediators activate tissue-resident immune cells to secrete anti-inflammatory factors and recruit immunosuppressive cells into tissues in attempt to counteract the inflammatory responses [10]. Given that the primary cause of inflammation cannot be resolved, it generates degenerative processes in tissues with aging [11]. The role of fibroblasts in this microenvironment is interesting since these cells can promote either pro-inflammatory or immunosuppressive processes to stimulate repair processes, such as fibrosis, in an attempt to maintain tissue homeostasis. I will examine the properties of myofibroblasts within tissues and the interactions of myofibroblasts with the cells of the immunosuppressive network. It seems that myofibroblasts play a significant role not only in the promotion of the aging process but also in the aggravation of age-related diseases.

## Plasticity and heterogeneity of fibroblasts

Fibroblasts are a diverse set of mesenchymal cells which originate from the mesodermal layer during embryogenesis [1, 2]. Fibroblasts possess a multilineage differentiation potential; e.g., in tissue injuries and remodeling states, they can serve as progenitor cells for many specialized mesenchymal cells, such as adipocytes, endothelial cells, pericytes, and osteoblasts. Interestingly, fibroblasts can also possess a pluripotency since adult fibroblast cells have been exploited in the generation of induced pluripotent stem cells (iPSC) [12]. Fibroblasts have also revealed a great diversity in their gene expression patterns with respect to their location within adult tissues and between different tissues [13, 14]. This positional memory seems to be driven by the epigenetic regulation of the *HOX* genes. Fibroblasts can also retain the memories of their past experiences, i.e., distinctive memories of previous inflammatory, metabolic, and mechanical stimuli [14]. These adaptive properties are crucial for the maintenance of tissue homeostasis since tissue-resident fibroblasts are the sensors of the tissue microenvironment and they have to be able to adapt to different insults in order to maintain tissue integrity. In addition, there are some specialized fibroblast populations which have tissue-specific functions. For example, the fibroblastic reticular cells in lymph nodes coordinate the crosstalk between immune cells, recruit immune cells into lymph nodes, and can even present antigens [15]. Moreover, the stromal bone marrow fibroblasts are the servant cells for the hematopoietic stem cells and progenitor cells in the regulation of hematopoiesis [16].

Single-cell transcriptional profiling studies have revealed the impressive heterogeneity in the properties of fibroblasts within normal tissues and between different tissues [17, 18]. Buechler et al. [18] reported an extensive study on the organization of fibroblast lineage across different mouse and human tissues both in the steady-state condition and in some diseases. They observed that there existed two universal subsets of fibroblast populations in the tissues of both normal mice and healthy humans. They suggested that these two major subgroups could act as a source population for the activation of more specialized fibroblasts in both the steady-state and diseased microenvironments. They also speculated that the tissue-resident fibroblasts of these universal subsets would be able to exhibit an inflammatory phenotype, whereas there existed six different clusters of fibroblasts, e.g., myofibroblasts, which represented a more persistent cellular state in perturbed tissues. Fibroblasts have also been categorized according to their specialized functional states, such as CAFs, fibrosis-associated fibroblasts (FAF), wound-associated fibroblasts (WAF), and aging-associated fibroblasts (AAF) [2]. Many single-cell transcriptional experiments have been conducted to characterize CAFs, FAFs, and WAFs [19–21]. These studies will be addressed later with respect to the immunosuppressive properties of the fibroblast subsets. Fibroblasts are commonly exploited in cell culture studies, especially in experiments examining cellular senescence. However, it should be recalled that fibroblastic cell lines, e.g., the NIH3T3 line, contain heterogeneous subclones which can be differentially expanded in distinct experimental treatments [22]. Moreover, fibroblasts which have been cultured on plastic plates or on collagen and gelatin-coated plates will switch into myofibroblast-like phenotypes attributed to the stiffness of the matrix [23].

**Fig. 2:**
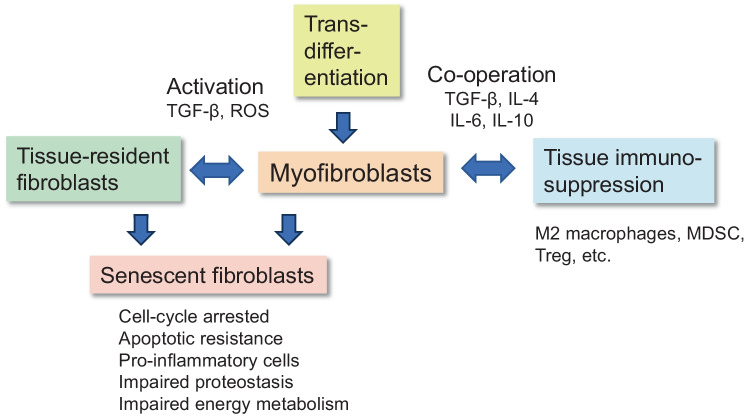
Myofibroblast differentiation and interaction with immunosuppressive cells in the promotion of an immunosuppressive microenvironment in aging tissues and age-related diseases. Cooperation with immunosuppressive cells can also promote the transdifferentiation of macrophages, monocytes, fibrocytes, adipocytes, pericytes, endothelial cells, epithelial cells, and smooth muscle cells into myofibroblasts. Tissue-resident fibroblasts can be activated into myofibroblasts in many pathological states. Tissue-resident fibroblasts and myofibroblasts can be converted into senescent fibroblasts in many pathological conditions. The common properties of senescent fibroblasts are listed. Abbreviations: IL, interleukin; MDSC, myeloid-derived suppressor cell; ROS, reactive oxygen species; TGF-β, transforming growth factor-β; Treg, regulatory T cell

In pathological conditions, there exist different cellular sources to account for the heterogeneity of fibroblasts within tissues. Tissue-resident fibroblasts can adapt to alterations in their microenvironment, e.g., to inflammatory changes and tissue wounds, or the presence of cancer cells which are able to edit the properties of local fibroblasts. Interestingly, the fate-mapping of cells has revealed that in pathological states, many cell types can become transdifferentiated into fibroblasts, most commonly into myofibroblasts (Fig. 2). For example, recruited monocytes and inflammatory macrophages can be transdifferentiated into myofibroblasts, e.g., as occurs in renal fibrosis [24]. The signaling via the TGF-β/Smad3 pathway is recognized as a common trigger for the transition of inflammatory macrophages into myofibroblasts during the generation of mouse kidney fibrosis [25]. Since adipocytes are mesenchymal-derived cells, they are an important source of myofibroblast transdifferentiation in the production of fibrosis in many tissues [26]. For instance, while the adipocyte–myofibroblast transition represents an efficient repair of skin wounds [27], it has also a significant role in dermal fibrosis and subcutaneous lipoatrophy [28]. Microvascular pericytes can also become transdifferentiated into myofibroblasts, e.g., in idiopathic pulmonary fibrosis [29]. Fibrocytes are the bone marrow (BM)-derived mesenchymal progenitor cells which are released into the circulation and recruited into affected tissues. Subsequently, the fibrotic or inflammatory microenvironment stimulates their differentiation into myofibroblasts [30]. Niu et al. [31] demonstrated that the differentiation capacity of circulating fibrocytes into myofibroblasts was significantly elevated in elderly people as compared to younger individuals. They reported that the age-related enhancement of differentiation competence was attributed to a more effective signaling through the IL-18/IL-18R1 pathway. Sueblinvong et al. [32] demonstrated that old mice possessed an increased level of fibrocytes in their BM as compared to younger mice, and accordingly, bleomycin treatment induced a stronger recruitment of fibrocytes into the lungs of aged mice.

It is also known that the transdifferentiated state of myofibroblasts can be reversed into their original phenotypes [33, 34]. For instance, Fortier et al. [34] demonstrated that PGE2 and FGF2 treatments were able to revert the myofibroblastic state of human lung fibroblasts. They reported that the dedifferentiation induced by PGE2 utilized the cAMP/PKA pathway, while FGF2 exploited the MEK/ERK pathway. There is clear evidence that the differentiation of myofibroblasts is under epigenetic regulation [35–37]. For instance, DNA methylation and noncoding microRNAs are the master regulators of myofibroblastic state, e.g., via the control of α-smooth muscle actin (α-SMA) expression in rat lung fibroblasts [35]. The resolution of tissue fibrosis can occur via different mechanisms involving either apoptosis or the dedifferentiation of myofibroblasts [38]. Interestingly, Kato et al. [39] demonstrated that myofibroblasts obtained from mice with idiopathic pulmonary fibrosis revealed an impaired capacity to undergo the dedifferentiation process. They also reported that fibrotic myofibroblasts displayed an increased resistance to apoptosis which was associated with an elevated expression of myoblast determination protein (MyoD). The MyoD factor can promote the differentiation of myofibroblasts by activating the expression of α-SMA protein in human fetal lung fibroblasts [40]. It seems that the transdifferentiation of diverse cell types to myofibroblasts highlights the exceptional plasticity of these cells and most probably increases the heterogeneity of fibroblast populations in pathological states.

The state of cellular senescence is one of the sources which increase the heterogeneity of fibroblast population in aged tissues as well as in idiopathic pulmonary fibrosis and systemic sclerosis [7, 41, 42] (Fig. 2). The hallmarks of senescent fibroblasts include, e.g., an irreversible cell-cycle arrest, apoptotic resistance, impaired proteostasis and energy metabolism, and the pro-inflammatory phenotype of senescent cells (Fig. 2). The inflammatory properties of senescent fibroblasts have been discussed in the “Fibroblasts act as immune regulators” section. Zou et al. [43] utilized single-cell transcriptomic technique to study age-related changes in cell populations of human normal skin. They demonstrated that the age-related variability of specific properties was significantly higher in the fibroblast population as compared to other cell types present in human skin. They revealed that the expression of α-SMA and many fibrous components of ECM was clearly downregulated in the fibroblast population of aged normal skin, whereas the expression of cytokines and other inflammatory mediators was strongly increased in old dermal fibroblasts. Several studies on systemic sclerosis have demonstrated that senescent fibroblasts accumulated into the sclerotic skin [42, 44]. The common biomarkers of senescence, such as cell-cycle inhibitors, certain collagen components, and inflammatory markers, were significantly upregulated in fibroblasts of the sclerotic skin. Oxidative stress, especially the NOX4-mediated processes, has a crucial role in the cellular senescence and fibrotic lesions in systemic sclerosis [45]. It is known that normal aged tissues and many age-related diseases display similar changes in the biomarkers of senescence as observed in senescent fibroblasts induced by replicative or damaging treatments in cell culture conditions; however, there are clear indications that cellular senescence in vitro does not represent the functional phenotype of fibroblasts within aged tissues attributed to the presence of tissue microenvironment [7].

## Fibroblasts act as immune regulators

Immune functions are not only limited to the cells of hematopoietic system such as myeloid and lymphoid cells, but also many structural cells of tissues can act as immune regulators; i.e., they are able to produce inflammatory mediators and modify the properties of immune cells. Krausgruber et al. [46] demonstrated that fibroblasts, epithelial, and endothelial cells of twelve mouse tissues displayed clearly cell-type-specific and tissue-specific crosstalk with immune cells. The fibroblasts present in many tissues revealed a significant expression of complement component 3 (C3), NOD-like receptors, and interleukin receptors, as well as the expression of ligands and receptors for immune checkpoint proteins and certain growth factors in a tissue-specific manner. Moreover, they reported that the infection of mice with lymphocytic choriomeningitis virus (LCMV) induced an enrichment of distinct genes in the fibroblasts present in many tissues, e.g., the genes involved in viral defense, cytokine production, and even antigen processing and presentation. Recently, Ngwenyama et al. [47] demonstrated that mouse cardiac fibroblasts expressed major histocompatibility complex type II (MHCII) protein in inflammatory models. They reported that these fibroblasts were able to take up and process antigens which subsequently were presented to CD4^+^ T cells via MHCII proteins. Selective responses of fibroblasts were expected considering their heterogeneous properties and diverse functions of different tissues and pathological states. The fibroblasts are a multifunctional cell type which possesses an impressive adaptation capacity to respond to the changes in their microenvironment. There is robust evidence that tissue-resident fibroblasts have a key role in the activation and suppression of immune responses in tissues [48, 49]. Fibroblasts are not only able to enhance and maintain inflammatory responses, but they can also trigger an immunosuppressive state in inflamed tissues.

It is known that activated fibroblasts can express both immune-interacting and tissue-remodeling properties [48]. Öhlund et al. [50] demonstrated that the CAFs isolated from mouse and human pancreatic cancers contained two mutually exclusive and reversible fibroblastic subtypes, i.e., the inflammatory iCAF population and the myofibroblastic myCAF subset. Their transcriptional profiles revealed that the iCAFs displayed an increase in the expression of many cytokines, chemokines, and some other inflammatory mediators as compared to quiescent fibroblasts, whereas myCAFs/myofibroblasts showed an upregulation of αSMA and collagen proteins. The expression of cytokines IL-6, IL-11, and LIF as well as chemokines CXCL1 and CXCL2 was the most extensively increased factors in the iCAF population. Subsequently, several single-cell transcriptomic studies have confirmed these results and revealed several other subgroups with novel properties, e.g., immunosuppressive properties (see the “Cancer-associated immunosuppressive myofibroblasts” section). Several later studies have revealed the presence of iCAF populations in other tumor types [19, 51, 52]. The antigen-presenting CAFs (apCAF) are the third subpopulation of fibroblasts commonly observed in tumor microenvironments [53]. The apCAFs express the proteins of the major histocompatibility complex class II (MHC II), but they are also able to stimulate the differentiation of immunosuppressive Tregs [53]. Recently, Bryce et al. [54] proposed a putative classification for CAFs containing also the subtypes of metabolic CAFs (meCAF) and the complement-secreting CAFs (csCAF), in addition to the iCAF, myCAF, and apCAF subsets. The activation of iCAFs from tissue-resident quiescent fibroblasts in tumor microenvironments has a crucial role in the progression of tumor growth. For instance, the iCAFs secrete many chemokines and colony-stimulating factors which stimulate the maturation of myeloid and lymphoid cells in the BM from which they will be recruited into tumor sites.

Although the role of CAFs as an enhancer of inflammation and tumorigenesis has been studied in detail, the function of fibroblasts in chronic age-related inflammatory states still needs to be clarified. It is known that cardiac fibroblasts are central players in shaping both the inflammatory and fibrotic processes taking place in myocardial postinfarction repair [55, 56]. Daseke et al. [55] described the transitions of fibroblasts after mouse myocardial infarction (MI); (day 1 MI) fibroblasts were pro-inflammatory and anti-migratory, (day 3 MI) fibroblasts were anti-inflammatory/immunosuppressive and pro-angiogenic, and (day 7 MI) fibroblasts were fibrogenic and anti-angiogenic. Moreover, Venugopal et al. [56] characterized the maturation phases after MI; (day 7 MI) fibroblasts were proliferative myofibroblasts and (day 28 MI) fibroblasts were highly fibrotic matrifibrocytes. These results indicate that the resident fibroblastic population can adapt to the requirements of the microenvironment, adopting either inflammatory, immunosuppressive, or fibrogenic properties. In fact, there exists an extensive bi-directional communication between tissue fibroblasts and the immune system, not only in the tumor microenvironment but also in many other inflammatory and fibrotic conditions [57–59]. Pro-inflammatory fibroblasts secrete cytokines, chemokines, and colony-stimulating factors which stimulate immune cells in damaged tissue; in addition, these inflammatory mediators can activate hematopoietic stem cells generating myeloid and lymphoid cells which will be recruited into inflamed tissues. Within tissues, activated inflammatory fibroblasts enhance the differentiation of myofibroblasts which also display immunosuppressive properties and are able to activate the immunosuppressive network, as described below [6, 60]. Moreover, in injured tissues, pro-inflammatory fibroblasts can trigger the transdifferentiation of some cell types into myofibroblasts. It seems that tissue-resident quiescent fibroblasts can become activated in tissue damage and they are able to express many classical immune properties which help in their cooperation with immune cells during the tissue repair and remodeling processes.

Human diploid fibroblasts have provided an excellent cell culture model when examining the properties associated with replicative senescence [7, 61]. Subsequently, it has been revealed that many other cell types also display signs of cellular senescence, either via excessive replication or the changes associated with harmful insults. Interestingly, Campisi and her collaborators revealed that senescent fibroblasts and many other senescent cells exhibited a pro-inflammatory phenotype, called the senescence-associated secretory phenotype (SASP) [9, 62] (Fig. 2). In cultured human fibroblasts, the most robust increases in terms of cytokine secretion were observed for GM-CSF, CXCL1/2, CXCL8, MCP1/2, MIP-1α, and IL-1α [9, 62]. These results indicated that the secretion of fibroblasts with SASP stimulated hematopoiesis in the BM and increased the recruitment of immune cells into aged/inflamed tissues. Subsequently, it has been demonstrated that cellular senescence not only appears in aged tissues, but this phenomenon is clearly evident in many age-related diseases, such as atherosclerosis, idiopathic pulmonary fibrosis, and systemic sclerosis [41, 42, 63, 64]. There seems to exist a substantial heterogeneity in the secretion profiles of senescent fibroblasts with respect to the experimental model being utilized, e.g., between the age-related and injury-associated senescent fibroblasts [65]. Single-cell studies of fibroblasts from aged tissues have also revealed a significant difference between aged fibroblasts with respect to their transcriptomic profiles in mouse heart [66] and human skin [67]. It is known that NF-κB signaling has a crucial role in the expression of pro-inflammatory mediators in senescent fibroblasts [68, 69]. Acosta et al. [69] demonstrated that the NLRP3 inflammasomes coordinated the secretory program in senescent human fibroblasts. There is convincing evidence that colony-stimulating factors, cytokines, and chemokines secreted by senescent fibroblasts not only activate the differentiation of tissue-resident fibroblasts into myofibroblasts but also stimulate the generation and differentiation of myeloid and lymphoid immune cells into immunosuppressive myofibroblasts. Lopez-Antona et al. [70] demonstrated that the senescence of myofibroblasts in culture was associated with a robust loss of myofibroblastic properties, e.g., the expression of α-SMA and collagens. They also revealed that the myofibroblastic phenotype of human fibroblasts was controlled by the NF-κB and Notch/TGF-β signaling pathways in a paracrine manner, i.e., Notch/TGF-β signaling enhanced fibrogenic properties, whereas NF-κB signaling stimulated inflammatory properties and decreased fibrogenic activity. Mellone et al. [71] reported that the senescence of human foreskin fibroblasts promoted a myofibroblastic differentiation which, however, expressed a reduced level of fibrotic proteins both in culture and the skin. The high fibrogenic activity encountered in senescent myofibroblasts was associated with an increased cancer incidence.

## Interactions of myofibroblasts with the immunosuppressive network

Given that tissue-resident fibroblasts have a crucial role in the maintenance of tissue homeostasis, it requires a close interaction between immune and non-immune cells [72–74]. This collaboration is emphasized in pathological conditions when the fibroblasts organize acute inflammatory responses and subsequently adapt to chronic inflammatory conditions. As discussed below, fibroblasts/myofibroblasts are key players in the generation and maintenance of immunosuppressive states, e.g., in the cancer microenvironment [6, 75, 76].

### Immunosuppressive network

Inflammatory responses associated with pathological conditions stimulate a compensatory anti-inflammatory response, thus preventing the expansion of detrimental inflammation [77–79]. In particular, the chronic presence of an inflammatory microenvironment evokes an immunosuppressive state which counteracts inflammatory responses in many age-related diseases, e.g., atherosclerosis, Alzheimer’s disease, cancer, and macular degeneration [80–83]. Inflammatory insults initially stimulate the expression and subsequently increase the secretion of a variety of cytokines, chemokines, and CSFs. These secreted factors not only control the phenotype of tissue-resident immune cells, such as macrophages and innate lymphoid cells, but also augment myelopoiesis in the BM [84]. For instance, CSFs and some chemokines trigger the generation of myeloid-derived suppressor cells (MDSC) which are released from the BM into the circulation and recruited into inflamed tissues [85]. There are two subgroups of MDSCs, i.e., the monocytic MDSCs (M-MDSC) and granulocytic MDSCs (G-MDSC), which are immunosuppressive cells although they have specific functions in pathological conditions [85]. MDSCs are very plastic cells which can be differentiated into certain other myeloid cells, e.g., macrophages, in inflammatory and fibrotic states. Activation of the immune system also promotes the generation of other immune cells in the BM, lymphoid organs, and also in affected tissues. The state of chronic inflammation modifies the phenotypes of immune cells, either by secreting anti-inflammatory factors, such as TGF-β, IL-4, and IL-10, or through the contact-dependent interactions, e.g., via inhibitory immune checkpoint receptors [86]. For example, the pro-inflammatory M1-type of macrophages can be converted into immunosuppressive M2 macrophages [87] and T cells into different types of regulatory T cells (Treg) [88]; i.e., immunosuppressive cells can augment the immunosuppressive properties of many immune cells inducing the so-called regulatory phenotypes. In addition to MDSCs and M2 macrophages, the immunosuppressive network includes also the regulatory B cells (Breg) and regulatory dendritic cells (DCreg), as well as the regulatory natural killer (NKreg) and type II natural killer T (NKT) cells. The properties and functions of the immunosuppressive network have been elucidated in detail in many extensive reviews [10, 85, 89, 90]. Moreover, we have reviewed the properties of immunosuppressive cells in association with the aging process [10, 91].

Immunosuppression is associated with several crucial beneficial responses in acute inflammatory states, but in chronic inflammatory conditions, it exerts many detrimental effects since it can inhibit the resolution of inflammation and thus prevent the repair process of tissue injuries [78, 90, 92, 93]. Activation of the immunosuppressive network reduces the functional activity of the immune system, especially that of adaptive immunity. The decline in the efficiency of the immune system with aging has been called immunosenescence [94]. Immunosenescence is also an important player in the pathogenesis of cancer and many age-related diseases [95, 96]. Interestingly, the activation of immunosuppressive cells, such as MDSCs, Tregs, and M2 macrophages, induces alterations in effector immune cells which are reminiscent of those encountered in immunosenescence [93, 97]. For instance, immunosuppressive cells (i) inhibit the proliferation of immune cells, (ii) prevent antigen presentation and antibody production, and (ii) reduce the cytotoxic activity of NK and CD8T cells and thus impair immune surveillance. Furthermore, it is known that CAFs suppress the function of NK cells and in this way impair the ability of the immune system to detect and destroy malignant cells [98, 99]. On the other hand, NK cells can kill the hepatic stellate cell-derived myofibroblasts and thus limit the severity of liver fibrosis [100]. The immunosuppressive armament contains many tools which suppress the function of immune cells, such as the secretion of reactive oxygen and nitrogen species (ROS/RNS) and anti-inflammatory cytokines, e.g., TGF-β and IL-10. These products not only have direct effects on immune cells, but they can also induce deteriorations in the functions of neighboring cells and thus induce tissue atrophy and fibrosis [11]. Fibrosis is most likely associated with the TGF-β-induced activation of myofibroblasts in stressed tissues. Immunosuppressive cells also repress the function of immune effector cells by enhancing the catabolism of arginine and tryptophan in inflamed tissues by increasing the synthesis of arginase 1 (ARG1) and indoleamine 2,3-dioxygenase 1 (IDO1) [11]. In this case, the immunosuppressive cells exploit the auxotrophy of many immune cells; i.e., these cells are unable to synthesize either arginine or tryptophan amino acids. Consequently, the deprivation of those two amino acids associated with the immunosuppressive state also impairs the protein synthesis and homeostasis in non-immune cells located in inflamed tissues.

### Crosstalk between myofibroblasts and immunosuppressive cells

Tissue-resident immunosuppressive myofibroblasts, i.e., the subsets of CAFs or activated fibroblasts in fibrotic and inflammatory states, are evidently a part of the immunosuppressive network and are able to cooperate with immune cells [6, 19, 59, 60, 101]. As stated above, inflammatory fibroblasts/myofibroblasts secrete several chemokines and CSFs, e.g., CCL2, CXCL12, and GM-CSF, which stimulate myelopoiesis in the BM. Increased myelopoiesis augments the generation and release of many myeloid cells, e.g., monocytes, MDSCs, and fibrocytes, which are subsequently recruited into the affected tissues. Interestingly, TGF-β has a key role in stress conditions; i.e., TGF-β secreted by immunosuppressive cells stimulates the differentiation of myofibroblasts, whereas TGF-β produced by CAFs/myofibroblasts induces the differentiation of MDSC, Tregs, and M2 macrophages [6, 60, 101] (Fig. 2). Meng et al. [24] reported that inflammatory macrophages were able to transdifferentiate into myofibroblasts in mouse kidney fibrosis. The depletion of the myeloid lineage attenuated the accumulation of myofibroblasts and accordingly reduced a severity of renal fibrosis. It is known that TGF-β exposure also triggers an alternative activation of M2 macrophages, i.e., the generation of the immunosuppressive M2 phenotype [102]. M2 macrophages secrete TGF-β and IL-10 cytokines in chronic diseases, and thus, they are not only able to induce myofibroblast differentiation but also activate several immunosuppressive cells [103]. For instance, Sheng et al. [104] demonstrated that the M2 macrophages induced the differentiation of tissue-resident fibroblasts into myofibroblasts through the secretion of TGF-β and IL-4 cytokines in human benign prostatic hyperplasia. They also reported that TGF-β treatment induced the differentiation through the Smad3 signaling pathway, whereas IL-4 utilized the STAT6/AKT/ERK axis. It does seem clear that the M2 macrophages are able to induce the differentiation of fibroblasts into myofibroblasts and thus promote fibrosis in pathological conditions [105, 106].

Tissue-resident fibroblasts/myofibroblasts also undertake a crosstalk with MDSCs and Tregs in the generation of the immunosuppressive microenvironment. Sun et al. [107] demonstrated that G-MDSCs promoted the age-related mouse cardiac fibrosis by activating myofibroblasts via the secretion of S100A8/A9 proteins. It is also known that peroxynitrite, e.g., a reactive radical produced by MDSCs [108], stimulated the differentiation of human embryonic lung fibroblasts into myofibroblasts [109]. Accordingly, Liu et al. [110] demonstrated that the amounts of MDSCs in the circulation were significantly increased in human idiopathic pulmonary fibrosis. They also revealed that MDSCs were able to promote the differentiation of mouse lung fibroblasts into myofibroblasts. Moreover, they reported that a deficiency of mouse MDSCs reduced the severity of lung fibrosis highlighting the crucial role of MDSCs in fibrotic diseases. However, the MDSC-induced myofibroblast differentiation seems to be dependent on the type of MDSCs or the experimental context since the M-MDSCs inhibited the myofibroblastic differentiation of mesenchymal stem cells [111]. Moreover, Pinchuk et al. [112] demonstrated that human colonic myofibroblasts enhanced the expansion of the FoxP3-positive Tregs and thus suppressed the symptoms of inflammatory bowel disease. Saxena et al. [113] revealed that Treg cells recruited into mouse infarcted cardiac muscle modulated the phenotype of fibroblasts; e.g., they reduced the expression of α-SMA and attenuated the contraction of fibroblast-populated collagen pads, thus combatting the myofibroblast-induced cardiac fibrosis. Accordingly, a depletion of Tregs aggravated the postinfarction inflammatory response. These few examples indicate that myofibroblasts collaborate with the cells of the immunosuppressive network, thus enhancing the development of an immunosuppressive microenvironment. The cooperation between tissue-resident fibroblasts and recruited immunosuppressive cells has been extensively studied in the initiation and progression of cancers.

### Cancer-associated immunosuppressive myofibroblasts

During the last decade, there has been dramatic progress made in our understanding of the functions of fibroblasts in tumorigenesis. Currently, it is known that CAF subpopulations are the architects of tumor initiation, growth, and spreading in a close cooperation with immunosuppressive cells [6, 59, 60, 101]. The multifunctional role of CAFs is based on their plasticity; several single-cell transcriptomic studies have revealed the extensive heterogeneity of CAF populations with respect to both the growth of tumors and their appearance in different types of tumors. Costa et al. [19] revealed that specimens of human breast cancer contained four different CAF subsets of which CAF-S1 displayed many myofibroblastic and immunosuppressive properties. For instance, the myCAF-S1 cells enhanced the recruitment of CD4^+^CD25^+^ T lymphocytes into cancers and subsequently induced their differentiation into the immunosuppressive FoxP3-positive Treg cells. The myCAF-S1 subgroup was characterized by its high expression of fibroblast activation protein (FAP), α-SMA, PDGFR, and CD73 [114, 115]. CD73 is an ecto-5’-nucleotidase enzyme which in pathological conditions converts AMP and ADP into adenosine. Extracellular adenosine is a potent enhancer of immunosuppression induced by Tregs and MDSCs [116, 117]. Subsequently, Kieffer et al. [118] characterized several subclusters in the myCAF-S1 subset in human breast cancer specimens. They observed that there existed a positive feedback loop between specific clusters and the immunosuppressive activity of Tregs, i.e., indicating a crosstalk between the TGF-β secretion by myCAFs and the induction of two immunosuppressive checkpoint proteins, PD-1 and CTLA4, in human Tregs. Moreover, myCAF-S1 cells were highly FAP-positive not only in human breast cancer [118] but also in many other cancers and pathological conditions [119]. Interestingly, Yang et al. [120] demonstrated that the FAP enzyme activated STAT3 signaling via the FAK-Src-JAK2 pathway in fibroblasts isolated from mouse hepatoma. They also reported that the induction of FAP stimulated the expression and secretion of the CCL2 chemokine which promoted the infiltration of MDSCs into mouse liver tumors and enhanced their growth.

It is known that there is a close metabolic interaction between CAFs and cancer cells and that this crosstalk enhances the growth of tumors [121, 122]. Interestingly, several studies have indicated that both cancer cells and myofibroblasts/CAFs exploit aerobic glycolysis in their energy metabolism, a process called the Warburg effect [123, 124]. In aerobic glycolysis, glucose is metabolized to lactate which has a crucial role in tumor growth. In fact, there is clear evidence that CAFs and cancer cells establish a reciprocal lactate shuttle which promotes cancer progression [124, 125]. It is now known that tumor-secreted lactate activates CAFs in diverse cancer microenvironments [126, 127]. Lactate is not only an endpoint metabolite, but it is also a signaling molecule that can control many immune processes; e.g., it can evoke immunosuppression and suppress inflammatory responses [128, 129]. For instance, Husain et al. [128] reported that lactate exposure stimulated the proliferation and activity of MDSCs in mouse pancreatic cancer cells. Accordingly, they revealed that lactate suppressed the cytotoxic activity of NK cells by inhibiting the expression of the activating NKp46 receptor in mouse NK cells. Lactate can induce the lactylation of histones and in this way enhance the immunosuppressive activity of immune cells; e.g., it increases the activity and proliferation of MDSCs and Tregs and enhances the M2 polarization of macrophages [130]. It seems evident that CAFs possess several mechanisms through which they can promote the development and maintenance of immunosuppressive microenvironment in tumors.

The plasticity of fibroblasts is able to shape various aspects of tumor growth, e.g., by promoting the proliferation and survival of cancer cells, increasing angiogenesis and ECM remodeling, and enhancing the metastatic spread of cancer cells [121, 131]. In fact, cancer cells seem to be able to educate CAFs not only to enhance their growth and metastasis but also to help them to escape from the immune surveillance by NK cells and cytotoxic T cells [98, 132]. Shortly, CAFs orchestrate an immunosuppressive state in the cancer microenvironment by recruiting immune cells into tumor sites and subsequently promoting their differentiation into regulatory phenotypes, such as MDSCs, Tregs, M2 macrophages, and tumor-associated macrophages (TAM). These immunosuppressive cells secrete a wide range of compounds, e.g., TGF-β and IL-10, which accordingly promote the immunosuppressive activity of CAFs. In addition, CAFs induce the expression of inhibitory immune checkpoint receptors or their ligands and in that way inactivate the functions of tumor-invading lymphocytes [133]. Currently, it is known that immunosuppressive CAFs can mount a crucial blockage impairing successful tumor immunotherapy. There are different strategies for the CAF-targeted anticancer therapies, e.g., inhibiting the function of immunosuppressive FAP and preventing the actions of TGF-β and IL-6 cytokines [6]. There are a number of extensive review articles depicting in detail the role of immunosuppressive CAFs in tumor progression and their importance in the disruption of successful cancer therapy [6, 60, 101, 134].

### Myofibroblasts associated with fibrotic lesions

Myofibroblasts are key players in the formation of fibrotic lesions; this is attributed to their role as a major source of the components of fibrous connective tissue [3, 5, 135]. Fibrosis is typically associated with tissue injuries, i.e., wound healing and acute scarring, but an excessive deposition of fibrous components within tissues can also be a reactive process associated with diverse pathological states involving chronic inflammation. Fibrosis can occur in all tissues, but more commonly, it appears in the lungs (IPF), myocardium (infarcts, aging), skin (systemic sclerosis, keloids), bone marrow (myelofibrosis), joints (arthrofibrosis), bowel (inflammatory bowel disease), vascular tissues (arthrofibrosis, systemic sclerosis), and internal organs (systemic sclerosis). TGF-β cytokine is a master regulator of the deposition of fibrotic components into fibrotic lesions [136]. In addition to TGF-β signaling, the Wingless (WNT) and Yes-associated protein 1 (YAP/TAZ)-mediated signaling pathways have also been implicated in the formation of fibrosis [137]. Currently, it is known that epigenetic pathways regulate both the progression and the maintenance of fibrosis, e.g., in systemic sclerosis and pulmonary fibrosis [36, 138]. There is robust evidence that the TGF-β and WNT signaling pathways enhance the differentiation of myofibroblasts and control their profibrotic state. However, several single-cell transcriptomic studies have revealed that there is a wide heterogeneity in the properties of fibroblasts and myofibroblasts present in fibrotic tissues [139, 140]. This is not surprising since in fibrosis myofibroblasts can have been differentiated from a variety of precursor cell types, such as macrophages, fibrocytes, tissue-resident fibroblasts, pericytes, smooth muscle cells, and endothelial cells.

There is an intimate crosstalk between myofibroblasts and immune cells during the progression of fibrosis [141–143]. This is attributed to the fact that TGF-β and several chemokines and cytokines are regulators in both myofibroblasts and many immunosuppressive cells. In brief, tissue injuries and the associated inflammatory responses involving the activation of myofibroblasts recruit immunosuppressive cells, such as regulatory lymphocytes (Treg, Breg) and myeloid cells (MDSC, M2 macrophages), which strive to counteract the pro-inflammatory responses. Consequently, these immunosuppressive cells secrete TGF-β and other cytokines which are potent enhancers of the differentiation and fibrotic activation of myofibroblasts; i.e., the immunosuppressive cells stimulate fibrosis in affected tissues. Subsequently, the initiation of the fibrogenic process increases the stiffness of the matrix, thus enhancing myofibroblast differentiation and fibrotic activity [144, 145]. For instance, Sheng et al. [104] demonstrated that M2 macrophages secreted IL-4, an anti-inflammatory cytokine, which induced the phenoconversion of fibroblasts into myofibroblasts in human prostatic hyperplasia. Birjandi et al. [146] demonstrated that the increased level of Tregs aggravated the bleomycin-induced mouse pulmonary fibrosis. Moreover, Liu et al. [110] reported that the levels of MDSCs and Tregs were increased in the circulation of IPF patients. In the mouse model of bleomycin-induced pulmonary fibrosis, they observed that MDSCs increased myofibroblast differentiation and enhanced the suppression of T cell proliferation. Liu et al. [110] also observed that immunosuppression was mediated via the B7H3 inhibitory checkpoint receptor in mouse MDSCs. Treatment of mice with anti-B7H3 antibodies inhibited the recruitment of MDSCs into fibrotic lungs and reduced severity of the pulmonary fibrosis.

While fibrosis can be a reversible process, it is recognized that its impaired resolution aggravates tissue fibrosis [39, 147]. The resolution of fibrosis is dependent on the elimination of the fibrogenic myofibroblasts and the degradation of the fibrotic ECM. There are studies indicating that myofibroblasts can be removed by apoptosis or modified into non-fibrotic cells through cellular senescence, dedifferentiation, and reprogramming. For instance, the apoptotic clearance of myofibroblasts is clearly declined in systemic sclerosis [135, 148]. It is known that mechanosensing via increased stiffness of ECM activates the Rho-associated kinase (ROCK) which subsequently increases the expression of anti-apoptotic BCL2 proteins and thus suppresses the elimination of myofibroblasts [135]. Moreover, an increased activity of PI3K/AKT pathway in systemic sclerosis inhibits the activity of pro-apoptotic BAX protein, thus enhancing the survival of myofibroblasts in systemic sclerosis. Currently, there is a debate on what role of the increased number of senescent myofibroblasts plays during the expansion of fibrosis; i.e., the senescent phenotype of myofibroblasts seems to limit fibrosis although on the other hand, senescence has been associated with a pro-inflammatory SASP which might augment compensatory immunosuppression and enhance the fibrotic activity of myofibroblasts via TGF-β exposure [63].

### Myofibroblasts in aging and age-related diseases

Fibrosis is a typical hallmark of the aging process in several tissues [4, 149, 150]. Age-related tissue fibrosis is associated with profound changes in the molecular components and physical structure of the ECM although there are many species- and tissue-specific differences [150–152]. For example, there is a decline in collagen synthesis, and thus, while the amount of collagen decreases with aging, conversely, the stiffness of ECM clearly increases due to an age-related increase in the level of collagen cross-linking. As described earlier, via a process called mechanosensing, the matrix stiffness regulates the differentiation of fibroblasts into myofibroblasts. For instance, Levental et al. [153] demonstrated that an increased amount of collagen cross-linking and greater stiffness of the ECM promoted the progression of mouse breast cancer. These modifications of the ECM increased integrin signaling and focal adhesions leading to enhanced tissue fibrosis. It is known that integrins, e.g., integrin α1β1, control myofibroblast differentiation in human tissues [154]. ECM and integrin signaling also regulate many immunological functions in aging tissues. For instance, Xing et al. [155] reported that an increase in matrix stiffness promoted the polarization of human macrophages into the immunosuppressive M2 phenotype. Increased substrate stiffness also enhanced the activity of human Tregs [156]. Moreover, matricellular proteins stimulated the immunosuppressive activity of MDSCs in human breast cancer [157]. These studies indicate that age-related increase in collagen cross-linking and matrix stiffness can promote the differentiation of myofibroblasts and thus create the condition for the development of an immunosuppressive microenvironment. Age-related fibrosis can also be attributed to a decline in the resolution of fibrosis. Kato et al. [39] reported that in many tissues, age-related fibrosis was connected to an increased resistance to apoptosis of myofibroblasts preventing their apoptotic cell death. It is recognized that resistance to apoptosis increases with aging and cellular senescence, thus promoting the aging process [158, 159]. It seems that the increased matrix stiffness of aged tissues both enhances the survival of myofibroblasts and augments the immunosuppressive microenvironment.

Age-related diseases are progressive disorders which are typically associated with chronic inflammation and frequently also with fibrotic lesions. Moreover, the immunosuppressive network is commonly activated in order to counteract the chronic inflammation. However, the interactions between myofibroblasts and immunosuppressive immune cells still need to be clarified although they both are involved in the pathogenesis of these diseases. Studies on many cancers and idiopathic pulmonary fibrosis, both of which are common age-related diseases, have revealed that there is a close interaction between myofibroblasts and immunosuppressive cells, as described above. Currently, it is known that the presence of myofibroblasts as well as M2 macrophages, Tregs, and MDSCs can be detected in several age-related diseases. For instance, the early phase of atherosclerosis is associated with the activation of tissue fibroblasts, i.e., the differentiation of the fibroblasts into myofibroblasts, which produce important profibrotic factors, like TGF-β and angiotensin II [160]. Myofibroblasts in atherogenesis can also originate from macrophages, endothelial cells, pericytes, and fibrocytes. Especially, myofibroblasts have a crucial role in the pathogenesis of arterial restenosis and remodeling processes [161, 162]. Inflammatory fibroblasts/myofibroblasts are involved in the recruitment of immunosuppressive cells into atherogenic lesions although the interactions between fibrogenic and immunosuppressive cells in the pathogenesis of this vascular disease still need to be clarified. However, it seems that Tregs and M2 macrophages promote anti-atherogenic functions, thus enhancing resolving processes in atherosclerosis [163, 164]. The myofibroblast population has also an important role in cardiac fibrosis in cardiovascular diseases [3], chronic kidney disease [165], age-related macular fibrosis and degeneration [166], and some autoimmune diseases, such as rheumatoid arthritis and systemic sclerosis [135, 148, 167]. For instance, in systemic sclerosis, several immune cells, such as monocytes, macrophages, neutrophils, and Th2/Th17 cells, are able to stimulate the differentiation of myofibroblasts and enhance their functions [135]. Immune cells secrete several cytokines, e.g., IL-4, IL-6, IL-10, and TGF-β, which induce the differentiation of myofibroblasts and thus aggravate tissue fibrosis in systemic sclerosis [135]. It is known that the immunosuppressive network is clearly activated in aging-associated chronic diseases, but it has still to be clarified how the interactions with myofibroblasts promote the pathogenesis of these diseases.

## Activation of the immunosuppressive network with aging and age-related diseases

### Activation of immunosuppressive network with aging

Common immune hallmarks of the aging process are chronic low-grade inflammation, increased immunosuppression, and a decline in the functional efficiency of the immune system [8, 10, 168–171]. A low-grade induction of inflammatory responses with aging has been observed in different tissues using diverse research approaches, such as transcriptomic and single-cell analyses [170, 172, 173]. Accordingly, an increased immunosuppression has been revealed in several experimental and clinical studies [10, 171, 174, 175]. Enhanced immunosuppression with aging is most likely an effect intended to counteract the presence of chronic low-grade inflammation. There is clear evidence that the aging process in humans and mice increases myelopoiesis in the BM and simultaneously induces the expansion of MDSCs in the circulation and within different tissues [91, 174, 176–178]. It has also been shown that MDSCs displayed improved immunosuppressive properties with aging, such as an enhanced suppression of T cell activity. In addition, the frequency of Tregs was upregulated with aging in the blood and many tissues [174, 179, 180]. It is also known that there is an increased presence of the immunosuppressive M2 macrophages in several mouse tissues with aging, e.g., in the bone marrow, spleen, and lungs [181]. There are many clinical consequences of the age-related increase in immunosuppression and immunosenescence; e.g., the cancer risk increases, vaccination efficiency decreases, and susceptibility to infections is enhanced, but conversely, transplantation tolerance is improved [171]. Currently, the role of myofibroblasts in the age-related degeneration of tissues needs to be clarified. There is abundant indirect evidence that myofibroblasts have significant roles in various aspects of the aging process, e.g., as a part of the activated immunosuppressive network.

### Immunosuppression associated with age-related diseases

As described above, a chronic inflammatory state has a crucial role in the development and maintenance of many common age-related diseases, such as cancer and cardiovascular diseases [182]. The presence of chronic local and systemic inflammation stimulates immunosuppressive responses which evoke immune deficiencies and thus promote premature aging. For example, there is convincing evidence that cancer survivors, even young patients, have an increased risk for showing signs of premature aging, e.g., osteoporosis, muscle atrophy, pulmonary fibrosis, and the characteristics of frailty [183–185]. It is known that tumorigenesis stimulates both local and systemic immunosuppression involving the activation of the immunosuppressive network [81] as well as the induction of CAFs. There are several other age-related chronic inflammatory states, e.g., chronic kidney disease (CKD), chronic obstructive pulmonary disease (COPD), and rheumatoid arthritis in which immunosuppression and immunosenescence are enhanced and as a result the aging process is accelerated [186–188]. These inflammatory diseases are also accompanied by many comorbidities. Moreover, the immunosuppression encountered in CKD and COPD is associated with an accumulation of the cells of the immunosuppressive network in the circulation and the diseases tissues [189–191]. Age-related diseases are also accompanied by an increased accumulation of myofibroblasts and fibrotic lesions. Tissue fibrosis with aging has been commonly associated with the state of chronic immunosuppression [192]. For instance, in idiopathic pulmonary fibrosis, the cells of the immunosuppressive network, such as MDSCs, Tregs, and M2 macrophages, have a crucial role in triggering the differentiation of myofibroblasts, and thus, they act as enhancers of tissue fibrosis [143]. The activation of the immunosuppressive network in cardiovascular diseases (CVD) induces an immunosuppressive state which has both beneficial and detrimental effects; e.g., the enhanced differentiation of myofibroblasts promotes harmful fibrosis in the cardiovascular system. While counteracting the chronic inflammation, it does seem that the immunosuppressive cells present in many age-related chronic diseases concurrently promote the appearance of the myofibroblast-driven fibrotic lesions.

## Are immunosuppressive myofibroblasts promoting the aging process?

### Enhancers of the aging process are inducers of myofibroblast differentiation

**Fig. 3:**
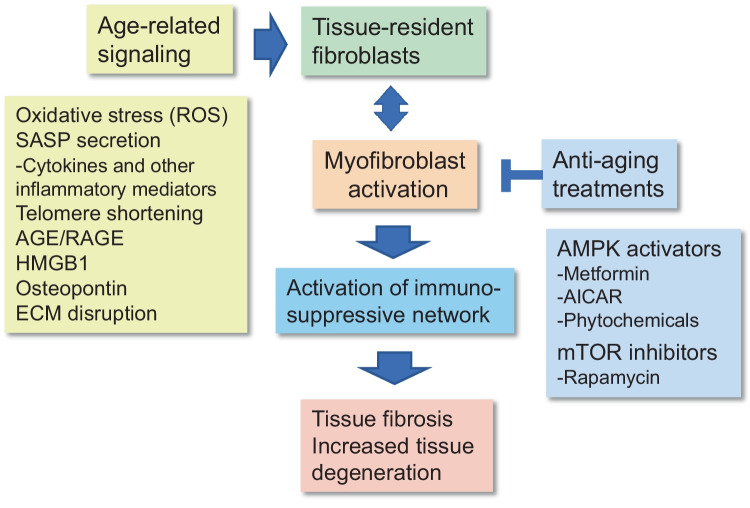
Age-related signaling stimulates the differentiation of myofibroblasts, whereas anti-aging treatments inhibit fibroblast differentiation. Age-related stimuli, e.g., oxidative stress and inflammatory mediators, can (i) induce the differentiation of myofibroblasts, (ii) enhance the recruitment of immunosuppressive cells into tissues, and (iii) create an immunosuppressive microenvironment in aged tissues. Accordingly, anti-aging treatments with metformin and rapamycin are able to suppress the differentiation of myofibroblasts in tissues and in this way inhibit the development of fibrotic lesions and prevent tissue degeneration. Abbreviations; AGE/RAGE, advanced glycation end-product/receptor of advanced glycation end-product; AICAR, 5-aminoimidazole-4-carboxamide ribonucleotide; DAMP, damage-associated molecular pattern; ECM, extracellular matrix; ER, endoplasmic reticulum; HMGB1, high-mobility group box 1 protein; mTOR, mammalian target of rapamycin; ROS, reactive oxygen species; SASP, senescence-associated secretory phenotype

The hallmarks of the aging process have been clarified although the primary cause driving the aging process still needs to be revealed [193]. Many of the hallmarks of aging, e.g., chronic low-grade inflammation, genomic instability, telomere attrition, mitochondrial dysfunction, and cellular senescence, can be induced by oxidative stress. In fact, oxidative stress induced by free radicals was one of the earliest postulated theories of aging [194]. Afterwards, the role of oxidative stress has been abundantly investigated as the cause underpinning the aging process and age-related diseases [195]. Reactive oxygen species (ROS) are generated by a wide variety of sources, such as macrophages and neutrophils in inflammatory states as well as mitochondrial respiration, NADPH oxidases (NOX), and ionizing radiation at the cellular level. ROS compounds have been demonstrated to be potent inducers of myofibroblast differentiation in diverse experimental setups [196–199] as well as enhancers of tissue fibrosis [200] (Fig. 3). Interestingly, the activation of TGF-β signaling induces the expression of the NOX4 enzyme via either the Smad3 or the Rho kinase pathways [198, 201]. Subsequently, the NOX4 enzyme stimulates ROS signaling which triggers the activation of myofibroblasts and promotes the generation of fibrotic lesions. For instance, Canugovi et al. [202] demonstrated that an increase in mitochondrial NOX4 expression with aging caused a stiffening of the aorta in mice and humans. Not only do ROS stimulate myofibroblast differentiation, but these reactive radicals can also activate immunosuppressive cells. For instance, the NOX enzyme-induced production of ROS stimulated the polarization of mouse M1 macrophages into M2 and tumor-associated macrophages (TAM) which are the two immunosuppressive phenotypes of macrophages [203]. Accordingly, the ROS produced by the NOX enzyme in rat and human macrophages induced the generation of immunosuppressive Tregs [204]. Nagaraj et al. [108] demonstrated that mouse MDSCs generated ROS and NO compounds which inhibited the T cell receptor (TCR) and thus suppressed the function of CD8^+^ T cells. Moreover, it is known that ROS compounds can activate the latent TGF-β1 complexes secreted by cells into the extracellular space and in that way enhance TGF-β signaling [205]. There is now convincing evidence that ROS compounds are signaling messengers in the immunosuppressive network and they can inhibit the function of immune cells and probably induce immune aging.

Oxidative stress also causes ROS-induced oxidation damages in both the intra- and extracellular spaces. For instance, these compounds can increase tissue stiffness and fibrosis with aging by promoting disturbances in the ECM and stimulating myofibroblast differentiation [206, 207]. As discussed above, stiffness and structural disturbances are major activators of myofibroblasts via mechanosensing of the changes in the ECM. ROS compounds also impair the integrity of genomic DNA, especially it is known that telomere dysfunctions promote the differentiation of human fibroblasts into myofibroblasts [208]. Razdan et al. [208] demonstrated that TGF-β exposure of human fibroblasts induced telomere dysfunction and the myofibroblast differentiation through the SMAD3/NOX4-dependent ROS production. Liu et al. [209] reported that the loss of the telomerase enzyme stimulated the differentiation of rat lung fibroblasts into myofibroblasts. Interestingly, telomere dysfunction has been recognized as a hallmark of aging and age-related diseases [210].

Given that fibroblasts can express both inflammatory and immunosuppressive properties, it does seem likely that these cells are involved in the regulation of the inflammaging process. It is known that age-related stresses, such as oxidative stress and endoplasmic reticulum (ER) stress, can trigger inflammatory responses and thus promote the aging process. Similar to oxidative stress, ER stress also stimulates the differentiation of myofibroblasts and enhances tissue fibrosis [211, 212]. There seems to exist diverse mechanisms which induce myofibroblast differentiation and fibrotic lesions although the major routes are mediated via the cooperation between TGF-β and NF-κB signaling, especially via the activation of NLRP3 inflammasomes [213–215]. It is known that the inhibitors of NF-κB and NLRP3 signaling are able to suppress the differentiation of myofibroblasts. For instance, TGF-β can stimulate NF-κB signaling which promotes the activation of the NLRP3 inflammasomes. There is also robust evidence that ROS compounds are potent activators of NLRP3 inflammasomes [216]. The mechanisms of the NLRP3-induced myofibroblast differentiation and generation of fibrotic lesions still need to be clarified although there are reports indicating that the IL-18 cytokine, a product of NLRP3 activation, enhances fibrosis in many experimental models [215, 217]. The activation of NLRP3 inflammasomes has been associated with the aging process and many age-related diseases [218]. Chronic inflammation is associated with several other factors which are able to induce myofibroblast differentiation and evoke fibrotic lesions, e.g., advanced glycation end-product and its receptor (AGE/RAGE) [219], high-mobility group box protein 1 (HMGB1) [220], CXC-type chemokines, e.g., CXCL12/CXCR4 [197, 221], and osteopontin protein [222] (Fig. 3). For instance, the osteopontin protein can (i) induce myofibroblast differentiation and promote fibrosis, (ii) control immunosuppression in tumors, and (iii) elicit many of the degenerative processes associated with aging and age-related diseases [222, 223]. To conclude, there is convincing evidence that enhancers of the aging process stimulate the differentiation of myofibroblasts and thus promote fibrosis both in aging tissues and age-related diseases.

### Anti-aging treatments suppress myofibroblast differentiation

Although the primary cause of aging is unknown, there have been promising results emerging from the anti-aging therapeutic experiments conducted in rodents with metformin and rapamycin [224, 225]. Metformin is an activator of AMPK signaling, and rapamycin is an inhibitor of mTOR activity. It is known that AMPK signaling activates autophagy by phosphorylating the Ulk1 protein at Ser317 and Ser777 [226]. Accordingly, mTORC1 phosphorylates the Ulk1 protein at Ser757, thus disrupting the interaction between the AMPK and Ulk1 proteins; i.e., both metformin and rapamycin stimulate autophagy. Autophagy is a crucial cellular cleansing mechanism, and it is known that its efficiency declines with aging disturbing healthy aging process and promoting many age-related diseases [227]. Interestingly, there is robust evidence that metformin exposure inhibits the differentiation of myofibroblasts and thus can prevent and even reverses tissue fibrosis in many experimental models [228–230] (Fig. 3). Two other activators of AMPK, i.e., AICAR and A-769662, also inhibited the TGF-β-induced myofibroblast differentiation of human primary mesangial cells. Chen et al. [231] reported that AICAR prevented the TGF-β-induced fibroblast–myofibroblast transdifferentiation and subsequently reduced the severity of kidney fibrosis in a mouse model of the ureteral obstruction. Several studies have revealed that AMPK signaling inhibits the TGF-β-induced activation of the Smad2/3 factor and alleviates its downstream functions, e.g., the expression of α-SMA and collagen proteins [228]. Several phytochemicals are also potent activators of AMPK signaling, and thus, they can inhibit myofibroblast differentiation and reduce the degree of tissue fibrosis. For instance, resveratrol suppressed the TGF-β-induced myofibroblast phenoconversion in human primary prostate and lung fibroblasts [232]. However, phytochemicals have commonly several cellular targets. Jiang et al. [233] have reviewed the studies investigating the role of AMPK signaling in the protection of fibrosis in different organs.

The mTOR kinase is a crucial factor in the regulation of the aging process and age-related diseases via its role in the control of protein synthesis, autophagy, and many metabolic processes [234]. There is clear evidence that rapamycin, an inhibitor of mTOR signaling, can inhibit fibrotic processes in different tissues [235, 236]. It is known that treatments with rapamycin represses the differentiation of myofibroblasts induced by TGF-β exposure in diverse cellular models [237] (Fig. 3). The activation of autophagy by rapamycin treatment seems to be a major mechanism accounting for the increase of health span by rapamycin. In addition to the regulation of mTOR and Ulk1 pathway, AMPK signaling also stimulates the activity of FoxO3, a recognized longevity factor [238]. Interestingly, Vivar et al. [239] reported that FoxO3a factor inhibited the TGF-β-induced differentiation of rat cardiac fibroblasts into contractile myofibroblasts. In conclusion, there is robust evidence emerging from work done with different experimental models indicating that the signaling pathways aggravating the aging process promote myofibroblast differentiation and age-related fibrosis, whereas anti-aging treatments suppress the differentiation of myofibroblasts.

## Conclusions

Currently, the tissue-resident fibroblasts have been overlooked players in the studies attempting to elucidate the mechanisms underpinning the aging process. Fibroblasts possess a number of properties which indicate that they have a major role in the aging process, e.g., (i) they possess an impressive cellular plasticity, (ii) they control the integrity of the ECM and the accumulation of fibrotic lesions, and (iii) they have multiple interactions with immune cells, and (iv) they are able to exhibit both the inflammatory and immunosuppressive phenotypes. The common hallmarks of aging involve a disruption of the ECM and the appearance of pro-inflammatory senescent cells which mediate a low-grade chronic inflammation. The interactions between fibroblasts and immune cells are especially interesting since fibroblasts are the sensors of diverse disturbances in tissue homeostasis. For instance, fibroblasts respond to acute insults, e.g., myocardial infarction, by adopting a pro-inflammatory phenotype, and thus, they are able to secrete cytokines and chemokines which activate tissue-resident immune cells and recruit immunosuppressive cells into the affected tissues. Interestingly, TGF-β and ROS compounds are not only the master inducers of myofibroblast differentiation, but they are also major messengers in the signaling between immunosuppressive cells, and thus, they promote the development of an immunosuppressive microenvironment in tissues. It does appear that the age-related low-grade inflammation promotes a compensatory immunosuppressive state in tissues by activating MDSCs, Tregs, and M2 macrophages. In view of the fact that myofibroblasts secrete TGF-β and some other immunosuppressive mediators, it seems that myofibroblasts collaborate with the immunosuppressive network which is not only activated with aging but especially in age-related diseases. By cooperating with myofibroblasts, immunosuppressive cells can regulate both the inflammatory state and repair processes underway in injured tissues. This might explain why immunosuppressive cells have such an important role in the development of the fibrotic processes occurring in the tissues of aged people.

## Data Availability

Not applicable.
